# *Cntn6* deficiency impairs allocentric navigation in mice

**DOI:** 10.1002/brb3.969

**Published:** 2018-04-20

**Authors:** Di Mu, Yiliang Xu, Tian Zhao, Kazutada Watanabe, Zhi‐Cheng Xiao, Haihong Ye

**Affiliations:** ^1^ Department of Medical Genetics and Developmental Biology School of Basic Medical Sciences Beijing Institute for Brain Disorders Center of Schizophrenia Capital Medical University Beijing China; ^2^ Department of Bioengineering Nagaoka University of Technology Nagaoka Niigata Japan; ^3^ The Key Laboratory of Stem Cell and Regenerative Medicine Institute of Molecular and Clinical Medicine Kunming Medical University Kunming China; ^4^ Department of Anatomy and Developmental Biology Monash University Clayton MEL Australia

**Keywords:** allocentric navigation, CNTN6, hippocampus, Morris water maze, spatial learning, spatial memory

## Abstract

**Introduction:**

CNTN6 is an immunoglobulin domain‐containing cell adhesion molecule that belongs to the contactin family. It is involved in the development of the nervous system. We aim to determine the effect of *Cntn6* deficiency on the allocentric navigation in mice.

**Methods:**

We recorded the travel distance and escape time of wild‐type and *Cntn6* mutant male and female mice in the Morris water maze task according to the protocol.

**Results:**

There was hardly any *Cntn6* expression in the hippocampus of postnatal day 0 (P0) mice, while obvious *Cntn6* expression was present in the hippocampal CA1 region of the P7 mice. During the acquisition period of Morris water maze task (Day 1 to 4), *Cntn6* ^*−/−*^ male mice failed to shorten the escape time to reach platform on the third day, while the travel distance to platform was not significantly different. There was no significant difference in both escape time and travel distance to the platform among all female subjects. In the probe trial test (Day 5), spatial memory of the female mutant mice was mildly affected, while *Cntn6* ^*−/−*^ male mice were normal. In the spatial relearning test (Day 7 to 10), *Cntn6* ^*−/−*^ male mice showed no difference in escape time to the platform compared to the wild‐type male mice, while *Cntn6* deficient female mice required shorter escape time to travel to the platform on day 7, day 8, and day 10.

**Conclusions:**

*Cntn6* is expressed in the developing hippocampus in mice. *Cntn6* deficiency affects spatial learning and memory, indicating that *Cntn6* plays a role in the development of hippocampus and affects allocentric navigation of the animals.

## 1 INTRODUCTION

Development of the central nervous system is dependent on the highly coordinated interactions between diverse cell types. Cell adhesion molecules (CAMs) are important signal molecules that mediate cell–cell and cell–extracellular matrix interactions in multiple neural developmental processes (Doving & Trotier, 1998; Schaal et al., 2003), including neuronal migration, neurite outgrowth, axon guidance, synaptogenesis, and synaptic connection (Dalva, McClelland, & Kayser, 2007; Geschwind & Levitt, 2007; Maness & Schachner, 2007; Murase & Schuman, 1999; Pardo & Eberhart, 2007; Rubenstein, 2011). Furthermore, CAMs may also function as receptors to regulate neuronal apoptosis and survival (Anderson et al., 2005; Naus et al., 2004).

Contactin‐6 (CNTN6), also termed NB‐3, is a member of the contactin family of immunoglobulin (Ig) domain‐containing cell adhesion molecules (IgCAMs). CNTN6 contains six N‐terminal Ig‐like and four fibronectin type III‐like (FNIII) domains and tethers to the cell membrane via a C‐terminal glycosylphosphatidylinositol (GPI)‐anchor (Maness & Schachner, 2007; Shimoda & Watanabe, 2009; Zuko et al., 2013). *Cntn6* has been identified as a candidate risk gene of multiple psychiatric disorders including autism spectrum disorders (ASDs), schizophrenia, bipolar disorder, attention‐deficit hyperactivity disorder, intellectual disability, and Tourette syndrome (Guo et al., 2012; Hu et al., 2015; Huang et al., 2017; Kashevarova et al., 2014; Kerner, Lambert, & Muthen, 2011; Nava et al., 2014; Oguro‐Ando, Zuko, Kleijer, & Burbach, 2017; Okbay et al., 2016; Pinto et al., 2010; Van Daalen et al., 2011), suggesting the necessity of CNTN6 in neural development.

In mice, *Cntn6* is exclusively expressed in the nervous system, such as cerebral cortex, accessory olfactory bulb, thalamus, and cerebellum (Huang, Yu, Shimoda, Watanabe, & Liu, 2012; Lee et al., 2000). However, the expression of *Cntn6* displays distinct patterns in different regions in the mouse brain. The level of *Cntn6* protein in the cerebrum reaches a maximum at P7 and thereafter declines to a constant low level in the adulthood (Huang et al., 2011; Lee et al., 2000). In contrast, the *Cntn6* mRNA level in the cerebellum and the hippocampus increases until the adulthood (Lee et al., 2000). Plenty of studies using null mutant mice indicate that *Cntn6* plays key roles in the developing and mature mouse brains (Mercati et al., 2013; Oguro‐Ando et al., 2017; Shimoda & Watanabe, 2009). In the visual cortex of one‐month‐old *Cntn6* ^*−/−*^ mice, alterations in the orientation of apical dendrites of pyramidal neurons in layer V was observed (Ye et al., 2008). *Cntn6* regulates neurite outgrowth in vitro*,* and this property was consistent with the finding that corticospinal tract formation was delayed in the *Cntn6* ^−/−^ mice (Huang et al., 2011, 2012; Mercati et al., 2013). Moreover, *Cntn6* contributes to glutamatergic synapse formation between parallel fibers and Purkinje cells during postnatal cerebellar development (Sakurai et al., 2009). In addition, behavioral studies have shown that *Cntn6*‐deficient mice display impaired motor coordination (Takeda et al., 2003).

In the hippocampus, a significant reduction in glutamatergic synapses was found in the *Cntn6*‐deficient mice in the postnatal stage (Sakurai, Toyoshima, Takeda, Shimoda, & Watanabe, 2010; Sakurai et al., 2009). Amila Zuko et al. found that *Cntn6* deficiency in the dentate gyrus (DG) may impair the fasciculation of mossy fibers that innervate pyramidal cells in the hippocampus (Cremer, Chazal, Goridis, & Represa, 1997; Heyden, Angenstein, Sallaz, Seidenbecher, & Montag, 2008; Montag‐Sallaz, Schachner, & Montag, 2002; Zuko et al., 2016). Some studies showed that F3/Contactin, another member of the contactin family, promotes hippocampal neurogenesis in adult mice (Mercati et al., 2017; Puzzo et al., 2013; Sakurai et al., 2009, 2010). These studies suggest that *Cntn6* may play an important role in the hippocampal development and function. However, the effect of *Cntn6* deficiency on hippocampal‐related behavior is still unclear.

In this study, we found that there was hardly any *Cntn6* expression in the hippocampus of P0 mice, but obvious *Cntn6* expression in the hippocampal CA1 region of P7 mice. Morris water maze task (MWM) was used to determine whether *Cntn6* deficiency in mice would affect allocentric navigation which involves hippocampus and its related brain structures. Our results suggest that deletion of *Cntn6* leads to functional deficiency of the hippocampus, especially the spatial learning ability in mice.

## 2 MATERIALS AND METHODS

### 2.1 Animal

*Cntn6*‐deficient mice (Takeda et al., 2003) were maintained on a 12‐hour light/dark cycle with ad libitum food and water in a specific pathogen‐free (SPF) animal facility at the Capital Medical University, China. All animal procedures were approved by the university's Committee for Animal experiments and conformed to the guidelines for the care and use of laboratory animals of the Chinese Society for Neuroscience.

*Cntn6* knockout mice were generated using 129/SVJ embryonic stem cells and then were backcrossed with C57BL/6J mice for more than 20 generations. In all experiments described in this article, homozygous and heterozygous mutants were compared with their wild‐type littermates.

### 2.2 Colorimetric detection of *LacZ* expression

*Cntn6* ^*+/−*^ mice at postnatal day 0 and 7 were perfused with PBS and then with 2% paraformaldehyde dissolved in PIPES, pH 6.9, containing 2 mM MgCl_2_ and 5 mM EGTA. Brains were removed and postfixed overnight at 4°C. The brains were then cryoprotected by incubation overnight in 20% sucrose containing 2 mM MgCl_2_. Floating sections (50 μm) were prepared using a cryostat. Sections were washed twice in PBS containing 2 mM MgCl_2_ and then incubated in PBS containing 2 mM MgCl_2_, 0.005% sodium deoxycholate and 0.01% NP‐40 for 10 min at 4°C. Colorimetric reaction was performed in the same solution containing 5 mM K_3_[Fe(CN)_6_], 5 mM K_4_[Fe(CN) _6_], and 0.05% 5‐bromo‐4‐chloro‐3‐indolyl–D‐galactoside (X‐gal) at 37°C overnight. The sections were washed, mounted, air‐dried and were counterstained with 0.5% neutral red to visualize the brain architecture.

### 2.3 Morris water maze task

Learning and memory tasks of adult mice (2–4 months) were assessed using a Morris water maze task according to previous reports (Petravicz, Boyt, & McCarthy, 2014; Schenk & Morris, 1985). The stainless steel circular pool (150 cm in diameter, 51 cm in depth) was filled with white opaque water maintained at 21 ± 1°C. The platform (10 cm in diameter) was submerged 1 cm beneath water surface. The locations of the starting points were identified using different colors and dimensions visual extra‐maze cues attached to the room walls and were kept consistent during each experiment. The pool was divided into four quadrants using a computerized tracking/image analyzing system (video camcorder coupled with computational tracking system: Coulbourn Instrument). During the acquisition training trails, the platform was placed in the middle of the northwest (NW) quadrant and remained in the same position. Subjects were placed pseudorandomly with their heads facing the pool wall into each of four starting locations (northwest, northeast, southeast, and southwest) for each of four daily acquisition training trials. Trials lasted 60 s or until the subjects mounted the platform with a 30‐min intertrial interval. On the first day (Day 1) of training, the subjects were manually placed on the platform and allowed to stand on it for 15–20 s if they did not find the platform after 60 s. The escape time, travel distance and mean velocity to reach the platform were recorded during the four‐day training. A probe trial to test reference memory was conducted on day 5. Subjects were placed into the opposite quadrant of the platform quadrant and allowed to swim during 60 s in the absence of the platform. The number of platform crossings, the number of target quadrant crossings, and the proportion of swimming time spent in four quadrants were recorded and analyzed.

The reversal task (relearning training trial) was performed from day 7 to day 10 exactly as the acquisition training protocol, while the hidden platform was placed in the opposite quadrant (southeast). The escape time, travel distance, and mean velocity to reach the platform were recorded. The subjects were blind to the genotypes.

### 2.4 Statistical analysis

A two‐way ANOVA followed by the Bonferroni posttest was used to analyze escape time to platform and travel distance. The results are displayed as mean ± standard error of the mean (SEM). Multiple *t* test followed by the Sidak–Bonferroni method was used to analyze the time in quadrant. A one‐way ANOVA followed by the Bonferroni posttest was used to analyze and obtain statistics of the entries to target quadrant.

## 3 RESULTS

### 3.1 Expression of *Cntn6* in the developing mouse hippocampus

**Figure 1:**
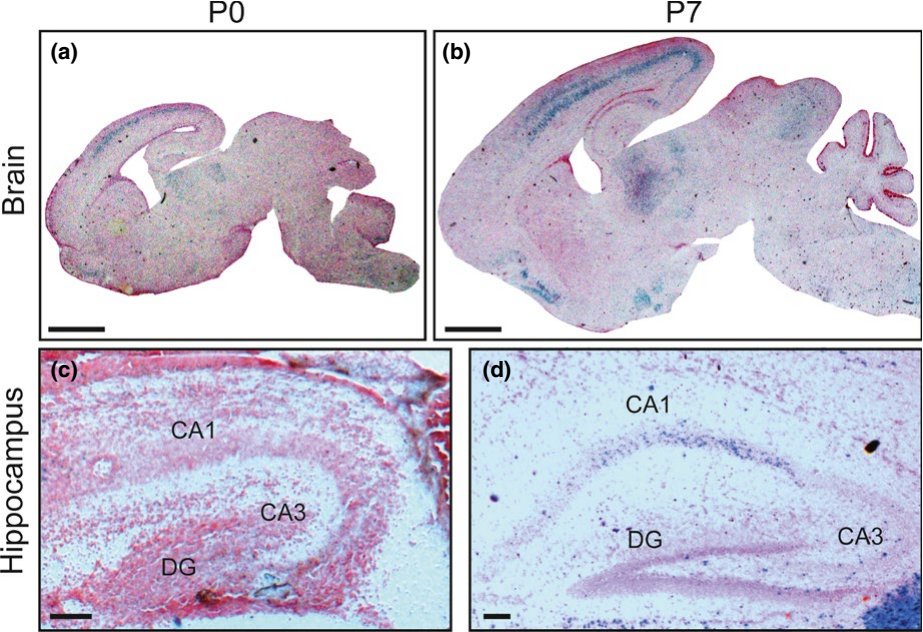
Expression of *Cntn6* in the developing mouse hippocampus. Localization of cells expressing *Cntn6* monitored by *LacZ* expression in the medial sagittal sections of the *Cntn6* ^*+/−*^ brains at P0 and P7. (c,d) Higher magnification of the hippocampus in (a,b). Scale bars, (a,b) 1 mm, (c,d) 0.1 mm

To assess the potential role of *Cntn6* in hippocampal development, the spatiotemporal expression of *Cntn6* was analyzed in the developing mouse hippocampus. The segment between initiation codon of the second exon and the Bgl I site in the second intron of the *Cntn6* gene was replaced by *LacZ* gene, so that the generated mutant mice were expected to produce β‐galactosidase instead of *Cntn6* protein. The *LacZ* gene expression was driven by the promoter of the *Cntn6* gene and accordingly reflected the expression of *Cntn6* (Takeda et al., 2003). We first examine the expression of the *LacZ* in the whole brain (Figure 1a,b) and hippocampus (Figure 1c,d) of P0 and P7 *Cntn6* ^*+/−*^ mice via X‐gal staining. The *LacZ* expression pattern was essentially the same as that observed in the *Cntn6* in situ hybridization previously reported by Lee et al. (2000). In the hippocampus of P0 mice, there was hardly any *Cntn6* expression in the CA1, CA3, and DG regions (Figure 1c). However, there was obvious *Cntn6* expression in the CA1 but not in the CA3 and DG regions of P7 mice (Figure 1d). These results were indicating that *Cntn6* is expressed in the developing hippocampus.

### 3.2 *Cntn6* deficiency affects spatial learning of male mice in the Morris water maze task

**Figure 2:**
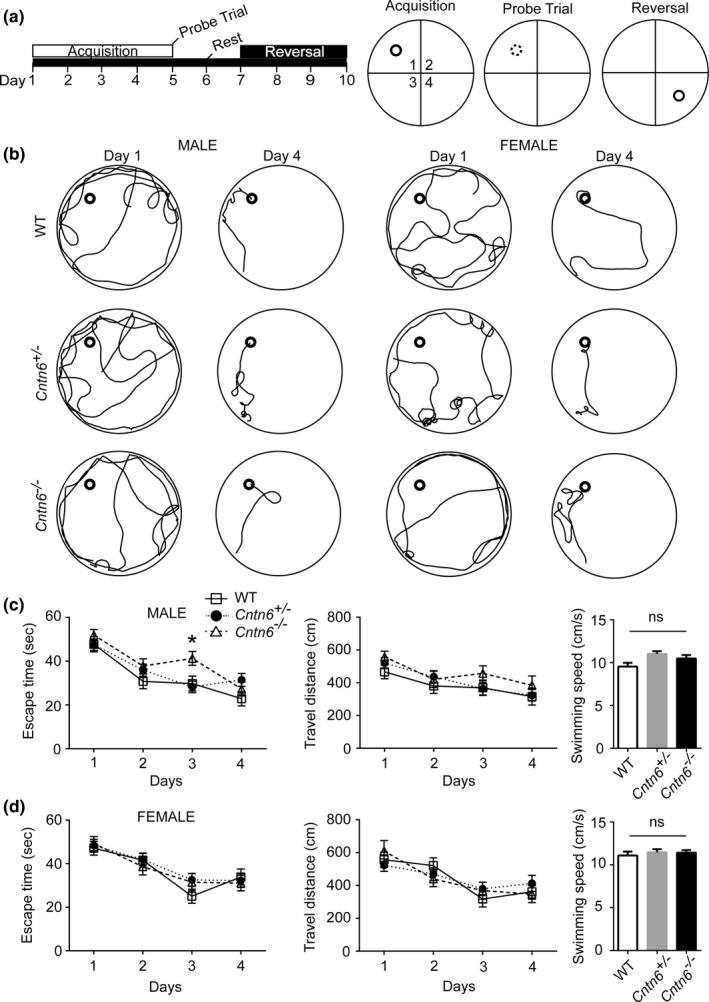
*Cntn6* deficiency affects spatial learning of male mice in the Morris water maze task. (a) A schematic representation of the Morris water maze training protocol. Mice were trained for 4 days to locate a hidden platform (acquisition trials). A probe trial was performed on the fifth day, when the platform was removed. The hidden platform was moved to the opposite quadrant during reversal training. (b) Representative traces of swimming plot in Morris water maze task. (c) Quantitative analyses of the Morris water maze. Performance of the *Cntn6* ^*−/−*^ male mice (2–4 months) in the spatial learning phases of the Morris water maze task, measured by escape time to platform. *n* = 10 (wild‐type, WT), 15 (*Cntn6* ^*+/−*^), 10 (*Cntn6* ^*−/−*^). Right panel, the swimming speed of male mice on the first day. (d) Performance of *Cntn6* ^*−/−*^ female mice in the spatial learning. *n* = 9 (WT), 12 (*Cntn6* ^*+/−*^), 8 (*Cntn6* ^*−/−*^). Data represent as mean ± SEM. Two‐way ANOVA followed by Bonferroni posttest for escape time and travel distance and One‐way ANOVA for swimming speed. *, *p *<* *.05; ns, not significant

The hippocampal structure plays an important role in spatial learning and memory. It has been reported that the length and area size of the suprapyramidal bundle (SPB) in the hippocampus were significantly increased in *Cntn6* ^−/−^ mice (Zuko et al., 2016). Here, we examined whether *Cntn6* deficiency affected hippocampus‐related behavior in the Morris water maze task. Over the 4‐day acquisition training period, all animals improved their ability to find the submerged platform by exhibiting shorter escape time and travel distance to the platform (Figure 2a,b). There was no significant difference in performance among all female subjects (Figure 2d). However, although *Cntn6* ^*−/−*^ male mice could swim as fast as wild‐type mice and willingly found a hidden platform, their escape time was significantly longer than their wild‐type and *Cntn6* ^*+/−*^ littermates on the third day (Figure 2c). No significant difference in escape time was detected on the fourth day in *Cntn6* ^*−/−*^ male mice (Figure 2c). These results indicated that spatial learning is mildly compromised in the *Cntn6* ^*−/−*^ male mice.

### 3.3 *Cntn6* deficiency affects the spatial memory of female mice, but not male mice

**Figure 3:**
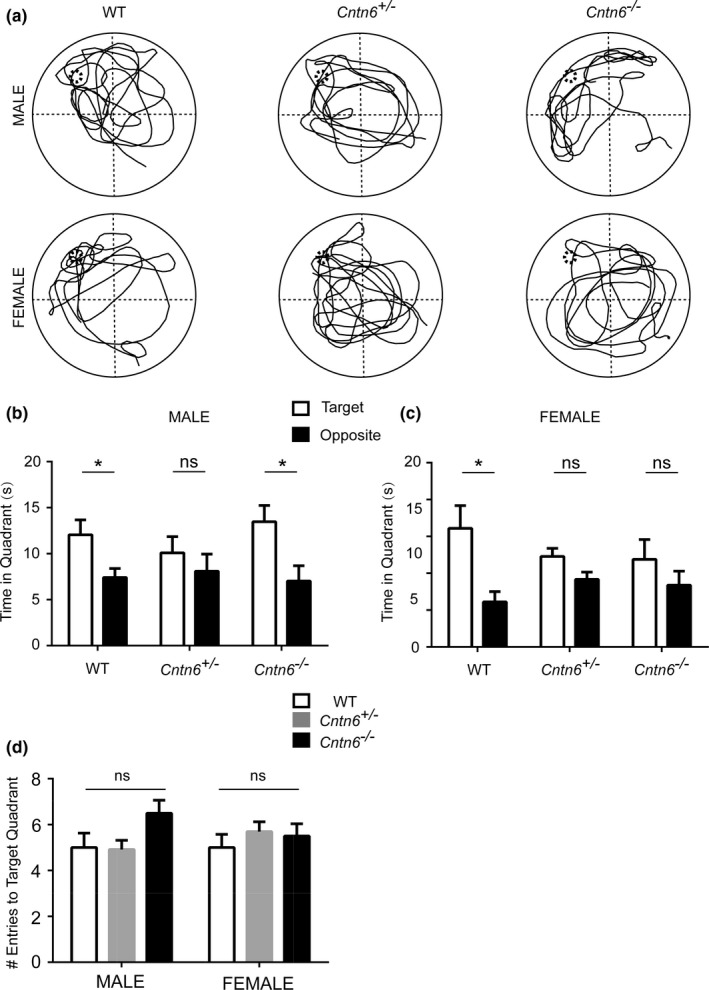
*Cntn6* deficiency affects spatial memory of female mice. (a–d), A probe trial of the Morris water maze. (a) Representative trajectories of WT,* Cntn6* ^*+/−*^, and *Cntn6* ^*−/−*^ mice during the probe trial. (b) The time male mice spent in the target quadrant and the opposite quadrant during the probe trial in which the target platform is removed. *n* = 10 (WT), 10 (*Cntn6* ^*+/−*^), 9 (*Cntn6* ^*−/−*^). Multiple *t* test followed by Sidak–Bonferroni posttest. (c) The time female mice spent in the target quadrant and the opposite quadrant during the probe trial. *n* = 7 (WT), 10 (*Cntn6* ^*+/−*^), 8 (*Cntn6* ^*−/−*^). Multiple *t* test followed by Sidak–Bonferroni posttest. (d) Number of entry to the target quadrant in the 60 s probe trial. Two‐way ANOVA. Data represent as mean ± SEM. *, *p *<* *.05; ns, not significant

After the 4‐day successive acquisition training period, we measured the time of movement of all the experimental groups in the 60‐second probe trial test on day 5 (Figure 3a). We calculated the time the mice spent in the target quadrant and the opposite quadrant after entering the pool in the last 40 s of the probe trial. Similar with the wild‐type male mice, *Cntn6* ^*−/−*^ mutant male mice spent significant shorter time in the opposite quadrant than in the target quadrant, indicating that the *Cntn6* deficiency has no serious effect on male mice's ability of recalling the previously learned spatial strategy (Figure 3b). Although *Cntn6* ^*+/−*^ and *Cntn6* ^*−/−*^ female mice also spent shorter time in the opposite quadrant, the change was not significant, (Figure 3c). We further analyzed the number of times the mice crossed the target platform location. There was no significant difference in the entries to target quadrant among all experimental subjects (Figure 3d). Together, these results indicated that *Cntn6* deficiency of leads to mild deficits in the spatial memory of female mice.

### 3.4 Improved spatial relearning in *Cntn6* deficient female mice

**Figure 4:**
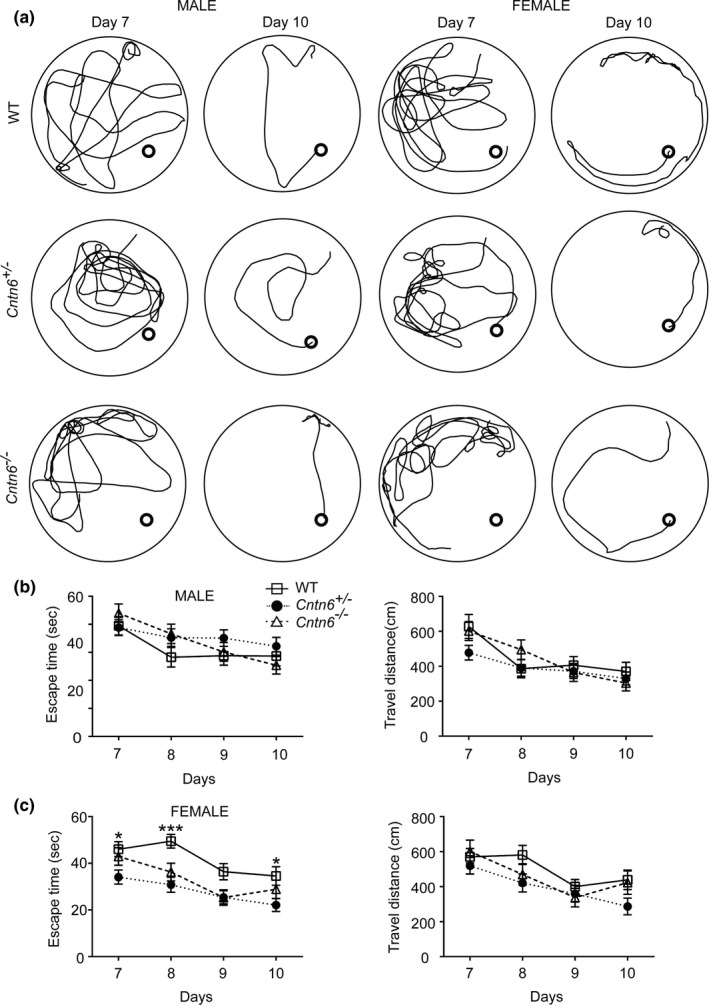
*Cntn6* deficiency improves spatial relearning of female mice. (a) Representative traces of swimming plot in Morris water maze reversal task. (b) Quantitative analyses of the Morris water maze. Performance of *Cntn6* ^*−/−*^ male mice (2–4 months) in the spatial relearning phase of the Morris water maze task, as measured by escape time to platforms. *n* = 10 (WT), 14 (*Cntn6* ^*+/−*^), 9 (*Cntn6* ^*−/−*^). (c) Performance of *Cntn6* ^*−/−*^ female mice in spatial relearning. *n* = 8 (WT), 12 (*Cntn6* ^*+/−*^), 8 (*Cntn6* ^*−/−*^). Data represent mean ± SEM. Two‐way ANOVA followed by Bonferroni posttest. *, *p *<* *.05; ***, *p *<* *.001

To investigate the effect of *Cntn6* deficiency on spatial relearning, we performed a reversal task in the Morris water maze. Mice were trained for 4 additional days (day 7 to day 10) with the hidden platform placed in the opposite quadrant (Figure 4a). There was no significant difference in travel distance between wild‐type and mutants mice in both sexes (Figure 4b,c). *Cntn6* ^*−/−*^ and *Cntn6* ^*+/−*^ male mice showed no difference in escape time to the platform in the reversal task (Figure 4b). Interestingly, compares with the wild‐type female mice, both *Cntn6* ^*+/−*^ and *Cntn6* ^*−/−*^ female mice spent shorter time to reach the platform, and the change was significant between the wild‐type and the *Cntn6* ^*+/−*^ female mice on day 7 (wild‐type vs. *Cntn6* ^*+/−*^, *p *=* *.031), day 8 (wild‐type vs. *Cntn6* ^*–/−*^, *p *=* *.0288; wild‐type vs. *Cntn6* ^*+/−*^, *p *=* *.0002), and day 10 (wild‐type vs. *Cntn6* ^*+/−*^, *p *=* *.0228) (Figure 4c). These results indicate that *Cntn6* deficiency improves spatial relearning in female mice.

## 4 DISCUSSION

Previous studies have shown that CNTN6 is important for the normal development and stability of the a few brain regions (Hu et al., 2015; Kashevarova et al., 2014; Lee et al., 2000; Sakurai et al., 2009). Here, we found that the expression of *Cntn6* in the hippocampal CA1 region increases during early postnatal stage, which is consistent with the data set provided by Allen Brain database (http://developingmouse.brain-map.org/gene/show/33165), suggesting that *Cntn6* is necessary for hippocampal structural formation and function. In the Morris water maze task, we found *Cntn6* ^−/−^ male mice failed to reduce the escape time to reach the hidden platform on day 3 of the acquisition trials. Interestingly, although female *Cntn6* mutant mice exhibited similar performance as the wild‐type mice in the acquisition trials, their spatial memory was mildly affected in the following probe trial. Moreover, female *Cntn6* mutant mice also showed a decreased escape time to reach the platform in the spatial relearning test.

The structural integrity of hippocampus is crucial for spatial learning and memory (Daugherty, Bender, Yuan, & Raz, 2016; Guderian et al., 2015; Penner & Mizumori, 2012). The so‐called “trisynaptic loop” in hippocampus conducts synaptic transmission and consists of three major excitatory pathways: perforant path (from entorhinal cortex to DG), mossy fiber (from DG to CA3), and Schaffer collateral (from CA3 to CA1) (Andersen, Bliss, Lomo, Olsen, & Skrede, 1969; Inoue & Watanabe, 2014; Kesner, Lee, & Gilbert, 2004; Knierim, 2015; Lee et al., 2017; Okada & Okaichi, 2009; Piatti, Ewell, & Leutgeb, 2013; Rolls & Kesner, 2006; Rongo, 2002). The CA1 region is also thought to help encode memory into a form that can be sent back to the entorhinal cortex via the subiculum for subsequent longer‐term spatial memory and consolidation, but not short‐term acquisition or encoding processes (Lassalle, Bataille, & Halley, 2000; Lee & Kesner, 2004; Rolls, 2015; Rolls, Dempere‐Marco, & Deco, 2013; Rolls & Treves, 1994; Rolls & Xiang, 2006; Treves & Rolls, 1992). We found that *Cntn6* is not expressed in the hippocampus of P0 mice, but is expressed in the CA1 region of P7 mice (Figure 1). Consistent with the expression pattern of *Cntn6*, the length and area size of mossy fiber projections in the SPB were significantly increased in the hippocampus of *Cntn6* ^−/−^ mice, indicating that *Cntn6* deficiency may impair the fasciculation of mossy fibers (Zuko et al., 2016). We therefore used the Morris water maze task to check whether the loss of *Cntn6* affects hippocampus‐regulated spatial learning and memory.

At first acquisition of spatial learning was evaluated via repetitive training during which the mice use distinct spatial cues to swim from the starting position to the submerged platform. On day 3 of the acquisition training trails, the *Cntn6* ^*−/−*^ male mice took longer time to find the hidden platform than the wild‐type male mice, indicating that *Cntn6* ^*−/−*^ male mice learn more slowly but catch up at a later stage of the acquisition training trials (Figure 2). This increase in escape time on day 3 in *Cntn6* ^*−/−*^ male mice is not due to impaired motor coordination as their swimming speed was comparable with the wild‐type male mice, and they performed equally well on day 1, 2, and 4 of the acquisition trials (Figure 2). After the acquisition training, a single probe trial was performed on day 5 with the platform withdrawn from the water tank to assess their spatial memory. The *Cntn6* ^*−/−*^ male mice performed similar as the wild‐type mice, while the spatial memory in female mutant mice was mildly compromised (Figure 3). Interestingly, in the relearning/reversal phase (day 7 to 10) when mice were forced to find the submerged platform at a different location, *Cntn6* ^*+/−*^ and *Cntn6* ^*−/−*^ female mice performed better than their wild‐type littermates (Figure 4), while no difference was detected in the male mice, suggesting that female *Cntn6* mutant mice are less perseverative for the previous acquisition platform location and are more readily to adapt to the changed contingencies.

Contactin family belongs to immunoglobulin (Ig) domain‐containing cell adhesion molecules (IgCAMs) and contains six members, CNTN1 (Contactin), CNTN2 (TAG‐1), CNTN3 (BIG‐1), CNTN4 (BIG‐2), CNTN5 (NB‐2), and CNTN6 (NB‐3) (Shimoda & Watanabe, 2009). CNTN6 is structurally and functionally similar to the other five family members. *CNTN4* and *CNTN6* followed by the close homologue of L1 (*CHL1*) are located on chromosome 3p25‐pter in the human genome (Kamei, Tsutsumi, Taketani, & Watanabe, 1998; Wei et al., 1998; Zeng et al., 2002). The deletion of this locus will cause 3p deletion syndrome with symptoms of microcephaly, growth retardation, intellectual disability, and distinctive facial features (Dijkhuizen et al., 2006; Fernandez et al., 2004, 2008). These three genes are closely located on chromosome 6p~ in the mouse genome and exhibit similar expression pattern. Thus, we speculate that the mild effect of *Cntn6* deficiency on learning and memory may be due to the compensational effects of other contactin family members for the in the *Cntn6* ^*−/−*^ brain.

Our results show that *Cntn6* mutant mice exhibit sexual difference in spatial learning and memory impairments. The selection of female mice was random and did not exclude the factors of the menstrual cycle. *Cntn6* ^*−/−*^ male mice show slower spatial learning, while female mutant mice may be compromised in long‐term memory retention. No sexual difference in hippocampus morphology or architecture has been discovered in the *Cntn6* mutant mice. Sex hormones are involved in the cognitive differences between men and women, and sex‐selective effects were also detected with regard to spatial learning and memory (Piber, Nowacki, Mueller, Wingenfeld, & Otte, 2018). Young males rodents also have an advantage in spatial learning in Morris water maze tasks (Brandeis, Brandys, & Yehuda, 1989). Male and female mice perform the same when they are 6 months old, suggesting that the sex difference in young animals may reflect a difference in maturation rate (Bucci, Chiba, & Gallagher, 1995). We also found that *Cntn6* ^*−/−*^ female mice have an advantage in spatial relearning during the reversal task compared with the wild‐type female mice. Reversal learning is a form of cognitive flexibility, an executive process that allows the adaptive modification of behavior in response to changes (Rygula, Walker, Clarke, Robbins, & Roberts, 2010). It has been reported that abnormal hippocampal structure leads to inflexible behaviors in women (Vilà‐Balló et al., 2017). We therefore speculate that the *Cntn6* deficiency may specifically increase cognitive flexibility in female mice.

In conclusion, *Cntn6* is expressed during postnatal hippocampal development. The absence of *Cntn6* affects hippocampal spatial learning and memory. However, its cellular and molecular mechanism need further study.

## CONFLICT OF INTEREST

The authors declare that they have no conflict of interest.
